# Combined LC-MS/MS and Molecular Networking Approach Reveals Antioxidant and Antimicrobial Compounds from *Erismadelphus exsul* Bark

**DOI:** 10.3390/plants11111505

**Published:** 2022-06-03

**Authors:** Morel Essono Mintsa, Elvis Otogo N’nang, Élodie Choque, Ali Siah, Justine Jacquin, Jerome Muchembled, Roland Molinié, Romain Roulard, Dominique Cailleu, Mehdi A. Beniddir, Cédric Sima Obiang, Joseph-Privat Ondo, Brice Kumulungui, François Mesnard

**Affiliations:** 1 UMRt BioEcoAgro 1158-INRAE, BIOPI, Université de Picardie Jules Verne, 1 Rue des Louvels, F-80000 Amiens, France; morel.essono.mintsa@etud.u-picardie.fr (M.E.M.); elodie.choque@u-picardie.fr (É.C.); roland.molinie@u-picardie.fr (R.M.); romain.roulard@u-picardie.fr (R.R.); 2 Laboratoire de Substances Naturelles, Université des Sciences et Techniques de Masuku, Franceville P.O. Box 943, Gabon; 3 UMRt BioEcoAgro 1158-INRAE, JUNIA, Équipe Métabolites Spécialisés D’origine Végétale, Institut Charles Viollette, F-59000 Lille, France; ali.siah@junia.com (A.S.); justine.jacquin@junia.com (J.J.); jerome.muchembled@junia.com (J.M.); 4 Plateforme Analytique, Université de Picardie Jules Verne, 33 Rue Saint Leu, F-80039 Amiens, France; dominique.cailleu@u-picardie.fr; 5 Équipe Chimie des Substances Naturelles BioCIS, CNRS, Université Paris Saclay, 5 Rue J.-B. Clément, F-92290 Châtenay-Malabry, France; mehdi.beniddir@universite-paris-saclay.fr; 6 Laboratoire de Recherches en Biochimie, Université des Sciences et Techniques de Masuku, Franceville P.O. Box 943, Gabon; cedricsima@gmail.com (C.S.O.); wansbop70@yahoo.fr (J.-P.O.); 7 Centre International de Recherches Médicales de Franceville P.O. Box 943, Gabon; kumulungui@yahoo.fr

**Keywords:** antimicrobial activity, antioxidant activity, de-replication, *Erismadelphus exsul*, molecular networks

## Abstract

*Erismadelphus exsul* Mildbr bark is widely used in Gabonese folk medicine. However, little is known about the active compounds associated with its biological activities. In the present study, phytochemical profiling of the ethanolic extract of *Erismadelphus exsul* was performed using a de-replication strategy by coupling HPLC-ESI-Q/TOF with a molecular network approach. Eight families of natural compounds were putatively identified, including cyclopeptide alkaloids, esterified amino acids, isoflavonoid- and flavonoid-type polyphenols, glycerophospholipids, steroids and their derivatives, and quinoline alkaloids. All these compounds were identified for the first time in this plant. The use of molecular networking obtained a detailed phytochemical overview of this species. Furthermore, antioxidant (2,2-diphenyl-1-picryl-hydrazylhydrate (DPPH) and ferric reducing capacity (FRAP)) and in vitro antimicrobial activities were assessed. The crude extract, as well as fractions obtained from *Erismadelphus exsul,* showed a better reactivity to FRAP than DPPH. The fractions were two to four times more antioxidant than ascorbic acid while reacting to FRAP, and there was two to nine times less antioxidant than this reference while reacting to DPPH. In addition, several fractions and the crude extract exhibited a significant anti-oomycete activity towards the Solanaceae phytopathogen *Phytophthora infestans* in vitro, and, at a lower extent, the antifungal activity against the wheat pathogen *Zymoseptoria tritici* had growth inhibition rates ranging from 0 to 100%, depending on the tested concentration. This study provides new insights into the phytochemical characterization and the bioactivities of ethanolic extract from *Erismadelphus* *exsul* bark.

## 1. Introduction

*Erismadelphus exsul* Mildbr (*E. exsul*), known as ‘Essang-Afane’ by local people (Fang) of northern Gabon, is a Gabonese medicinal plant. Its bark is used in folk Gabonese medicine to treat several microbial diseases, such as urinary tract infections, bronchitis, pneumonia, and sexually transmitted diseases—especially HIV opportunistic diseases [1]. *E. exsul* belongs to the family Vochysiaceae, which is commonly distributed across America and tropical Africa [2]. Indeed, Vochysiaceae is an important plant family in the neotropical plant formation [3]. Generally, plants belonging to this family are known for their numerous pharmacological properties, such as the case of *E. exsul*. However, despite being well known, *E. exsul* has not been the subject of any phytochemical or biological studies. Nevertheless, a few studies have been conducted on the Vochysiaceae family. For example, Weniger and coll. showed that several species of the *Vochysia* genus are used by traditional communities in South America to alleviate conditions related to inflammation, such as skin wounds, asthma, and pulmonary congestion [4]. The work of Franco and coll. also demonstrated the antioxidant and anti-glycation capacity of plant extracts from the Vochysiaceae family [5]. In addition, Dornelo and coll. reported that some hyphomycetes (fungi) could live in association with plants belonging to this plant family [6]. The literature studies reported here show the importance of searching for bioactive compounds in plants from the Vochysiaceae family, specifically *E. exsul*. Until now, the step of screening and identifying bioactive compounds from a plant extract was often very time consuming and tedious, as it consisted of purifying, testing, and individually identifying several compounds from a rich extract. In this context, new innovative bioinformatics approaches, such as molecular network (MN) and in-silico fragmentation tools, are currently being developed to make the search for molecules of biological interest less tedious [7,8]. Here we propose a de-replication strategy based on combining MN and liquid chromatography coupled with tandem mass spectrometry (HPLC-MS/MS) data to rapidly target and identify chemical constituents present in the ethanolic extract of *E. exsul*. The de-replication information was then collected by MN, which compares all MS/MS spectra of studied compounds and groups them according to their spectral similarity, thus allowing the visualization of all structurally related molecules within the same cluster and structurally different molecules distributed in different clusters [9]. This approach thus provides new ways to navigate the metabolome of biological samples by providing key information about the analogies between detected metabolites [10]. The main interest of molecular networks is that they can be used to exploit numerous MS/MS spectra without prior knowledge of the chemical composition of the samples, and the data obtained can be directly correlated to antimicrobial and antioxidant activity.

## 2. Results

One of the major focuses of the modern discovery of new compounds based on natural products is to apply an efficient strategy for the evaluation of secondary metabolites essential for human use. In this work, we demonstrate the usefulness of a new approach, molecular networking combined with LC-MS/MS, which is applied to the ethanolic extract of *E. exsul* in order to identify bioactive specialized metabolites of this extract.

### 2.1. Metabolite Profiling of E. exsul Determined via LC-MS/MS

**Figure 1:**
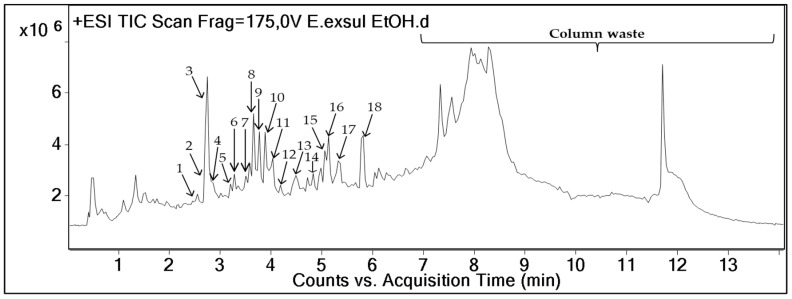
Total ion chromatogram (TIC) in positive ion mode of the ethanolic extract of *Erismadelphus exsul* obtained on an Agilent 6530 Q/ToF (Scan rang *m/z* 100–1700). The number above each peak corresponds to the peak numbers in Table 1.

LC-HRMS/MS analyses of the crude extract and fractions of *E. exsul* were performed in a positive ionization mode using the data-dependent acquisition mode. A TIC chromatogram of the crude extract is presented in Figure 1. Next, an auto-MS^2^ was carried out in which the most predominant MS^1^ ions are selected for MS^2^ fragmentation. A manual inspection of the resulted MS/MS spectra led to the putative identification of chemical constituents of the crude extracts and fractions of *E. exsul*; they are listed in Table 1.

Known and unknown compounds were annotated using databases such as: Dictionary Natural Product (https://dnp.chemnetbase.com/, accessed on 5 May 2022) [11,12], Computer software review: Scifinder (https://scifinder-n.cas.org, accessed on 5 May 2022), and PubChem (https://pubchem.ncbi.nlm.nih.gov/compound, accessed on 5 May 2022). As shown in Table 1, a total of 18 compounds were putatively identified. These metabolites were reported to belong to eight (8) families of natural compounds: cyclopeptide alkaloids, isoflavonoids and flavonoids (polyphenols), glycerophospholipids, esterified amino acids, steroids and their derivatives, and quinoline alkaloids

**Table 1:** Tentative identification of compounds from crude extract of *E*. *exsul* by LC−ESI-MS/MS in the positive ion mode.

Peak	RT (min)	*m/z* [M+H]^+^	Molecular Formulas [M]	MS^2^	Ions Tentative Identification	Confidence Level
1	2.571	477.250	C_27_H_32_N_4_O_4_	477.24, 460.74, 359.33, 265.95, 186.09, 120.08	Unknown	4
2	2.707	562.302	C_31_H_39_N_5_O_5_	562.30, 477.24, 378.18, 174.09, 134.06, 120.08	Mauritine F [13]	3
3	2.745	576.319	C_32_H_41_N_5_O_5_	576.31, 477.24, 378.18, 174.09, 134.06, 120.08	Mauritine A [13]	3
4	2.885	592.313	C_32_H_41_N_5_O_6_	592.31, 477.24, 378.18, 174.09, 134.06, 120.08	Mauritine A *N*-oxide [13]	3
5	3.088	433.113	C_21_H_20_O_10_	283.06, 313.07, 121.02, 165.01	Vitexin	3
6	3.206	599.330	C_30_H_38_N_12_O_2_	336.21, 458.27	Unknown	4
7	3.282	313.086	C_17_H_12_O_6_	315.08, 244.37, 272.80, 312.92, 103.07	Aflatoxin B2	3
8	3.282	579.171	C_26_H_28_O_15_	579.17, 152.01, 285.07, 460.87	Rhoifolin	3
9	3.661	439.357	C_30_H_46_O_2_	393.35, 339.15, 313.07	Ganodermenonol	3
10	3.774	453.336	C_30_H_44_O_3_	329.10, 264.23	Kulactone	3
11	4.646	322.23	C_19_H_31_NO_3_	322.23, 163.063, 288.198	8-Dihydroantidesmone [14]	3
12	4.987	306.243	C_19_H_31_NO_2_	290.98, 296.48, 178.086, 163.063	8-Deoxoantidesmone [14]	3
13	5.138	398.265	C_20_H_35_N_3_O_5_	306.24, 194.11	Unknown	4
14	5.365	320.222	C_19_H_29_NO_3_	278.24, 220.59, 222.11, 234.13, 163.063	Antidesmone [14]	3
15	5.782	256.133	C_16_H_17_NO_2_		Unknown	4
16	5.061	445.212	C_27_H_28_N_2_O_4_	283.09, 398.26, 194.11	Asperglaucide	3
17	5.782	507.228	C_32_H_30_N_2_O_4_	327.23, 256.13	Asperphenamate	3
18	6.123	317.196	C_16_H_28_O_6_		2-Bornanol (1*S*,2*R*)-*O*-d-glucopyranoside	3

The confidence level of compound annotations was performed with respect to the guidelines published by the Metabolomics Society [15]. Level 0: Unambigous 3D structure: isolated, pure compound, including full stereochemistry; Level 1: Confident 2D structure: uses reference standard match or full 2D structure elucidation; Level 2: Probable structure: matched to literature data or databases by diagnostic evidence; Level 3: Possible structure or class: most likely structure, isomers possible, substance class or substructure match; Level 4: Unkown feature of interest.

Among the cyclopeptide alkaloids, Mauritine F (2), Mauritine A (3), and Mauritine A *N*-oxide (4) at *m/z* 562.302 [M+H]^+^, *m/z* 576.319 [M+H]^+^, and *m/z* 592.313 [M+H]^+^, respectively, were putatively identified. Compounds 2, 3, and 4 were previously isolated by Han and coll. [13] from the stems of *Ziziphus apetala*. An analysis of the exact masses of the three alkaloids showed a mass difference of -14 Da between Mauritine F and Mauritine A, indicating a loss of one methylene (CH_2_) by Mauritine A; and a difference of +16 Da between Mauritine A and Mauritine A N-oxide, indicating one more oxygen than Mauritine A. Furthermore, the three compounds share the same MS/MS fragment ions 120.08 [M+H]^+^; 134.06 [M+H]^+^, and 174.09 [M+H]^+^, which were previously described by Kapadia and coll. [16]. Based on the literature, most cyclopeptide alkaloids differ only in their substituents in the asymmetric C34 position. Cyclopeptide alkaloids from epimeric pairs, wherein the two epimers can be transformed into each other, has the C34 position configured as (*R*) or (*S*) [13]. The available MS and MS/MS data do not allow us to assign with any certainty the epimer pairs present in the EtOH extract of *E*. *exsul.*

### 2.2. MS/MS-Molecular Networking-Based Dereplication

**Figure 2:**
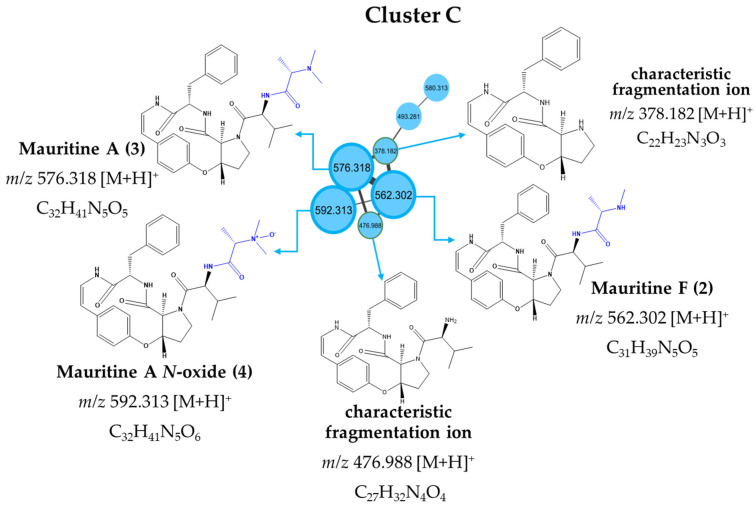
Putative annotation of cluster C with three cyclopeptide alkaloids compounds.

In addition, we also performed an analysis of the crude extract and fractions of *E*. *exsul* using the molecular network (MN) approach by the GNPS website (http://gnps.ucsd.edu, accessed on 5 May 2022) [17]. All obtained HRESIMS/MS spectra were preprocessed via MZmine2 [18] software following the feature-based molecular networking workflow [19]. These arrayed MS/MS spectra were then searched in an automated batch mode against the spectral libraries hosted by GNPS and the results were annotated as part of this de-replication strategy. A re-examination of the molecular network that had previously been obtained from the organization of the LC-MS/MS data of the crude extract and fractions of *E. exsul* revealed the presence of a total of 585 precursor ions visualized in Cytoscape 3.51 [20] as nodes in the molecular map, which included 8 clusters (node ≥ 2) and 346 single nodes (Supplementary Materials Figure S1). An exploration of the relative contributions of peak areas at nodes in the network revealed that the compounds were grouped based on structural similarities and three main clusters emerged: Cluster A corresponded to steroids and derivatives (red), Cluster B grouped glycerophospholipids (purple), Cluster D displayed quinoline alkaloids (blue), and Cluster C, of particular interest to us, was composed of seven nodes, three of which were at *m/z* 576.319, 562.302, and 592.313 (previously annotated as Mauritine A, Mauritine F, and Mauritine A *N*-oxide, respectively). The presence of characteristic ions at *m/z* 378.182 and *m/z* 476.988 in cluster C provided additional information on the identity of the above compounds [13,16] (Figure 2). Based on chemotaxonomic data, the distribution of cyclopeptide alkaloids shows that they are mainly present in Rhamnaceae (Genus *Ziziphus*) [21], Malvaceae (Genus *Melochia*) [22,23], and Euphorbiaceae (Genus *Antidesmone*) [24], and all Angiosperms (Dicotyledons) evolved in the Rosideae as well as the Vochysiaceae family of *E. exsul*. Finally, polyphenols such as Vitexin at *m/z* 433.113, Formononetin at *m/z* 269.083, the glycerophospholipid on the example of 1,2-Dilinoleoyl-sn-glycero-3-phosphocholine at *m/z* 784.588, terpenoids such as Sarmentoside B at *m/z* 663.463, and quinoline alkaloids such as Antidesmone at *m/z* 320.222, 8-Deoxoantidesmone at *m/z* 306.243 and 8-Dihydroantidesmone at *m/z* 322.238 were also annotated (Supplementary Materials Figure S2). It is important to highlight the fact that some compounds detected in this plant are described in fungi so far. This is the case of Asperphenamate, Asperglaucide, Formononetin, and Aflotoxin B2. The most certain hypothesis until now that explains this strong presence of the molecules of the fungal type in this plant are found in the work done in Brazil by Dornelo and coll. (2017) [6]. Indeed, they showed plants of the Vochysiaceae family, of which *Erismadelphus* is a member, have the ability to live in association (symbiosis) with hyphomycetes. Given the similarity in terms of climate (equatorial climate) between Gabon and Brazil, it seems important to consider this hypothesis without rejecting the fact that these molecules can come from the plant since the harvests were made on several sites. In order to assess the likelihood of a possible contamination of the samples, thorough molecular biology investigations will be performed in due course to get more insight into the chemical diversity of this plant.

The results show that the combination of molecular networks and LC-HRMS/MS data greatly improves the efficiency of the identification of chemical components in the targeted plant extracts. About seventeen compounds were putatively identified in the ethanolic extract of *E. exsul* bark (Table 2). The MN approaches also allowed us to support and confirm the conclusions from the interpretation of the MS/MS data for the three cyclopeptide alkaloid derivatives that we had previously identified. This network was carried out in a strategy of exploration of the metabolites present in the crude extract and fractions of *E. exsul*.

### 2.3. Antioxidant Activity

The antioxidant activity of crude extract and ethanolic fractions of *E. exsul* was evaluated by DPPH and FRAP methods using Trolox as a standard and ascorbic acid for comparison. Both methodologies react to different antioxidant mechanisms, including free radical scavenging for DPPH and iron reduction for FRAP. Overall, the crude extract and fractions obtained from *E. exsul* showed a better reactivity to FRAP than to DPPH (Table 3). The *E. exsul* fraction was two to four times more antioxidant than ascorbic acid while reacting to FRAP, whereas it was two to nine times less antioxidant than this reference while reacting to DPPH. These results are in agreement with the way *E. exsul* peel was used in traditional medicine. Indeed, ingestion in the form of herbal tea could allow for better assimilation of the metabolites and thus their passage through the blood system (where iron is located) than another type of traditional use such as cataplasm (where radical scavenging activity will be more efficient). Focusing on the crude extract responsiveness to both tests may seem surprising. Regarding FRAP, the antioxidant property of the crude extract is equivalent to those of fractions F4 to F8, and four times more antioxidant than ascorbic acid. This could be explained by a threshold effect or by the presence in those fractions of the same metabolite that reacts to FRAP. Regarding DPPH, the antioxidant property of the crude extract is 20 times lower than those of fractions F4 to F8, and 50 times less antioxidant than ascorbic acid. This result can be interpreted in three ways: (i) an antagonistic effect of metabolites present in the crude extract and separated in the fractions regarding free radical scavenging activity; (ii) the presence in the crude extract of a compound inhibiting radical scavenging activity of the compounds from fraction 3 to fraction 8; and less probably, (iii) nevertheless it cannot be excluded that the quantity and/or proportion of each metabolite in the crude extract led to this weak antioxidant activity. It has been widely demonstrated that polyphenols have a great capacity to scavenge free radicals and to chelate transition metals such as iron [25]. The high reactivity in the FRAP assay can be linked to the presence of several flavonoids and isoflavonoids such as Formononetin, Biochanin A, Rhoifolin, and Vitexin. Indeed, many studies have shown the strong antioxidant activity of Formononetin [26,27], Rhoifolin [28], and Vitexin.

These results are in agreement with those obtained by R.R Franco and coll. (2019) who showed strong antioxidant activities of several plants belonging to the Vochysiaceae family [5]. In fact, they are even more interesting because all of the activities observed by Franco and colleagues (2019) are lower than the antioxidant capacity of ascorbic acid regarding the FRAP test, while the extract and fractions obtained from *Erismadelphus exsul* are four times more antioxidant than ascorbic acid, suggesting a putative interest for industrial valorization such as nutraceutical potential.

### 2.4. Antimicrobial Assay

**Figure 3:**
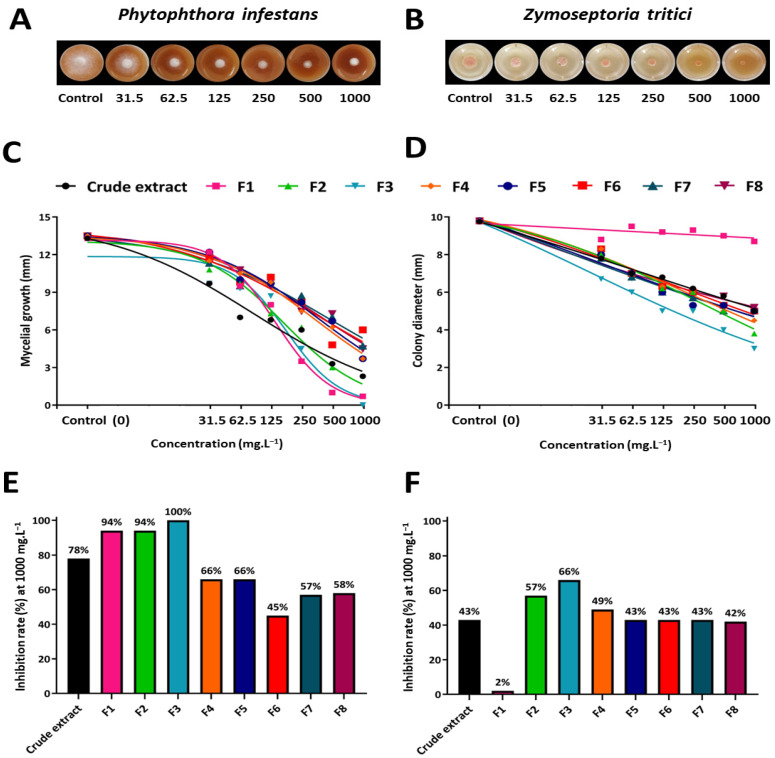
Antimicrobial activity of crude ethanolic extract and fractions from *Erismadelphus exsul* bark on *Phytophthora infestans* (**A**,**C**,**E**) and *Zymoseptoria tritici* (**B**,**D**,**F**) at three and eleven days after 12-well microplate inoculation with the pathogens, respectively. (**A**,**B**) illustration of the in vitro growth of *P. infestans* (**A**) and *Z. tritici* (**B**) obtained on V8 and PDA mediums, respectively, amended or not with different concentrations of the fraction F3. (**C**,**D**) Dose-response curves obtained for *P. infestans* (**C**) and *Z. tritici* (**D**) using the crude extract and fractions at different concentrations. (**E**,**F**) Percentage of inhibitions scored for *P. infestans* (**E**) and *Z. tritici* (**F**) using the crude extract and fractions at the highest tested concentration of 1000 mg·L^−1^. The X-axis graduation in both (**C**) and (**D**) subfigures is presented in Log2 of the tested concentrations.

The in vitro antimicrobial activity of *E. exsul* bark was measured on both *Phytophthora infestans,* an oomycete responsible for late blight on numerous Solanaceae such as tomato and potato, and *Zymoseptoria tritici*, a fungus responsible for *Septoria tritici* blotch of wheat. Two independent experiments with three biological replicates were performed for each pathogen and both experiments provided similar results (Supplementary Materials Table S1). Overall, close patterns of antimicrobial activity were observed for all fractions and the crude extract on each pathogen (Figure 3A). Dose-response curves revealed that all fractions, as well as the crude extract, inhibit the mycelial growth of *P. infestans*, especially at the highest tested concentration of 1000 mg·L^−1^ (Figure 3A,C). The most active fraction was F3, with 100 % mycelial growth inhibition at 1000 mg.L^−1^ (Figure 3E). Regarding *Z. tritici*, the fractions and the crude extract showed lower overall antimicrobial activities when compared to those obtained for *P. infestans* (Figure 3B,D). No fraction was able to totally inhibit the fungal growth, even at the highest tested concentration (Figure 3F). Interestingly, F3, the most effective fraction at 1000 mg·L^−1^ on *P. Infestans*, was also the most active one at this concentration on *Z. tritici* (Figure 3E,F). However, F1, one of the most active fractions on *P. Infestans*, displayed no activity on *Z. tritici* (Figure 3C–F). The difference in activity highlighted among the two targeted phytopathogens can be explained by the fact that the compounds responsible for the anti-oomycete activity occur in higher concentrations in the obtained fractions when compared to those responsible for the antifungal activity. On the other hand, this difference in the activity between *P. infestans* and *Z. tritici* might be explained by the physiology and the wall composition of the two species. Indeed, the composition of the wall plays a major physiological role in the exchanges between the intra- and extracellular environment. In fungi, the presence of chitin, glucans, and other polymers favors the maintenance of the flexibility of the wall, thus preventing certain molecules from penetrating the cell and reaching their cellular target [29]. This may, in part, explain the lower activity of molecules present in the extract and fractions of *E. exsul* on *Z. tritici*. Although several previous works have shown significant antimicrobial activities of a wide range of botanical extracts against both pathogens [30,31], this is the first report highlighting the antimicrobial activity of *E. exsul* extract against *P. infestans* and *Z. tritici*. The objective being to search for the molecules involved in these activities, the most active fractions were analyzed by mass spectrometry and the molecules of interest were identified and targeted using the molecular network approach. Thus, it was shown that cyclopeptide alkaloids (Mauritine A, Mauritine F, and Mauritine A *N*-oxide) and quinoline alkaloids (8-Deoxoantidesmone, 8-Dihydroantidesmone, and Antidesmone) are partly responsible for the antimicrobial activity of *Erismadelphus exsul* bark (Supplementary Materials Figures S3 and S4). This is consistent with several studies that have shown the antimicrobial activity of cyclopeptide and quinoline alkaloids. Among them, the work of Panseeta and coll. showed that cyclopeptide alkaloids have strong antimicrobial activities, especially against some fungi, mycobacteria, or protozoa [32]. Also, a study by Cretton and coll. showed that quinoline alkaloids exhibit antifungal activity against *candida albicans* [33]. In general, alkaloids play an important role in biological structures, they are known for their high antimicrobial power [34].

## 3. Materials and Methods

### 3.1. Plant Materials

Based on an ethnopharmacological survey of medicinal plants used in Gabonese traditional medicine, fresh plant samples were collected from the forests of Woleu-Ntem province in northern Gabon: (2°36′0″ N, 12°4′0″ E) (0°43′0″ N, 11°37′60″ E) in August 2018. Fresh bark from the trunks of *E*. *exsul* was cut using pruning shears, labeled, and transported in a UV-resistant plastic bag. Botanical identification was confirmed by Prof. Henri Paul Bourobou Bourobou and Mr. Raoul Niangadouma of the Institute of Pharmacopoeia and Traditional Medicine (IPHAMETRA) in Libreville. An herbarium reference specimen (M.E.M 001) has been deposited at the National Herbarium of Gabon (Libreville).

### 3.2. Extraction and Fractionation

The dried and pulverized barks of *E*. *exsul* (1500 g) were extracted by stirred maceration with 5 L of 70% ethanol at room temperature for 24 h. The resulting crude EtOH extract was vacuum filtered and concentrated by rotary evaporation to 3.64 g of an ethanolic fraction. All this residue was fractionated by flash chromatography on an 80 g Reveleris^®^ Grace Silica PF-50SIHC-F0080 cartridge (Rheinland-Pfalz, Germany) using a gradient of CH_2_Cl_2_, thereby increasing the proportions of MeOH (100:0 to 0:100). The flow rate was fixed at 60 mL/min. Separation by flash chromatography yielded fractions based on their chromatographic profiles. Fractions of a similar nature were grouped by TLC thin layer chromatography, which were analyzed by HPLC-QTOF-HRES in order to localize the potential new compounds.

### 3.3. Data Dependent LC-HRMS^2^ Analyses

HPLC-QTOF-HRES analyses were achieved by coupling the LC system to a hybrid quadrupole time-of-flight mass spectrometer Agilent 6530 (Agilent Technologies, Massy, France) equipped with an ESI source, operating in positive ion mode. Source parameters were set as follows: capillary temperature at 320 °C, source voltage at 3500 V, and sheath gas flow rate at 10 L.min^−1^. The divert valve was set to waste for the first 3 min. MS scans were operated in full-scan mode from *m/z* 100 to 1700 (0.1 s scan time) with a mass resolution of 11 000 at *m/z* 922. A MS^1^ scan was followed by MS^2^ scans of the three most intense ions above an absolute threshold of 5000 counts. The selected parent ions were fragmented with one collision energy fixed at 45 eV and an isolation window of 1.3 amu. The calibration solution containined two internal reference masses (purine, C_5_H_4_N_4_, *m/z* 121.050873, and HP-921 [hexakis-(1H,1H,3H-tetrafluoropentoxy)phosphazene], C_18_H_18_O_6_N_3_P_3_F_24_, *m/z* 922.0098). A permanent MS/MS exclusion list criteria was set to prevent oversampling of the internal calibrant. LC-UV and MS data acquisition and processing were performed using MassHunter Workstation software (Agilent Technologies, Massy, France).

### 3.4. Feature-Based Molecular Networking Parameters

The MS^2^ data files were converted from the .d (Agilent) standard data-format to .mzXML format using the MSConvert software, which is part of the ProteoWizard package [35]. All .mzXML were then processed using MZmine 2 v32 [18]. The mass detection was realized by keeping the noise level at 3.0 E3. The ADAP chromatogram builder was used using a minimum group size of four scans, a group intensity threshold of 3000, a minimum highest intensity of 4000, and *m/z* tolerance of 0.01 Da (or 20 ppm) [36]. The ADAP wavelets deconvolution algorithm was used with the following standard settings: S/N threshold = 8, minimum feature height = 3000, coefficient/area threshold = 8, peak duration range 0.02–2 min, and RT wavelet range 0.02–1.2. MS^2^ scans were paired using an *m/z* tolerance range of 0.03 Da and RT tolerance range of 1.0 min. Isotopologues were grouped using the isotopic peaks grouper algorithm with an *m/z* tolerance of 0.005 Da (or 15 ppm) and a RT tolerance of 0.3 min. Peak alignment was performed using the joint aligner module ***(****m/z* tolerance = 0.005 Da (or 15 ppm), weight for *m/z* = 2, weight for RT = 2.0, and absolute RT tolerance 0.3 min). The peak list was gap-filled with the same RT and *m/z* range gap filler module (*m/z* tolerance of 0.005 Da (or 15 ppm)). Next, the .mgf spectral data file and its corresponding .csv metadata file (for RT and areas) were exported using the dedicated “Export to GNPS-FBMN” built-in option.

### 3.5. Molecular Network Analysis

A molecular network was created using the online workflow at GNPS (http://gnps.ucsd.edu, accessed on 5 May 2022) with a parent mass tolerance of 0.02 Da and an MS/MS fragment ion tolerance of 0.02 Da. A network was then created where edges were filtered to have a cosine score above 0.65 and more than 6 matched peaks. Further edges between two nodes were kept in the network if and only if each of the nodes appeared in each other’s respective top ten most similar nodes. The spectra in the network were then searched against GNPS spectral libraries. All matches kept between network spectra and library spectra were required to have a score above 0.6 and at least 6 matched peaks. The molecular networking data were analyzed and visualized using Cytoscape software (ver. 3.9.0) [20].

### 3.6. Antioxidant Assay

The Ferric Reducing Power (FRAP) assay was performed according to the method of Benzie and Souche [37] with slight modifications. The working FRAP reagent was produced by mixing 300 mM of acetate buffer (pH 3.6), 10 mM TPTZ (2,4,6-tripyridyl-s-triazine) solution, and 20 mM FeCl_3_ in a 10:1:1 ratio just before use. Trolox (Sigma-Aldrich) was used as a standard. A 750 µM stock solution (diluted in ethanol) was prepared for Trolox. Then, serial dilutions were performed to obtain a range from 50 µM to 375 µM. After this, 950 µL of FRAP solution was combined with 50 µL of each standard dilution or its appropriately diluted sample. Each sample was done in triplicate and incubated at 37 °C for 30 min. The absorbance was read at 593 nm using a UV spectrophotometer (Anthelie Advanced, SECONAM).

### 3.7. 2,2-Diphenyl-1-picryl-hydrazyl-hydrate (DPPH•) Assay

DPPH free radical scavenging assay was adapted from Brand-Williams and coll. (1995) [38]. A 100 µM solution of DPPH was diluted in absolute ethanol. A 1 mM stock solution (diluted in ethanol) was prepared for Trolox. Then, serial dilutions were performed to obtain a range from 5 µM to 25 µM. After this, 1950 µL of DPPH solution was combined with 50 µL of each standard dilution or the appropriately diluted plant extract. Each sample was done in triplicate and incubated at room temperature for 20 min. The absorbance was read at 517 nm using a UV spectrophotometer (Anthelie Advanced, SECONAM).

### 3.8. Antimicrobial Assays

The anti-oomycete activity of the crude extract and fractions was evaluated according to the method described by Banso and coll. (1999), with slight modifications [39]. V8 medium was prepared by autoclaving at 121°C and cooled to 45 °C. Then, crude extract and fractions were prepared at a concentration of 100 mg·mL^−1^ in dimethyl sulfoxide (DMSO) and then added to the medium to have a range of six concentrations (1000, 500, 250, 125, 62.5, and 31.5 mg·L^−1^). Two milliliters of each mixture were poured into sterile 12-well microplates and allowed to solidify. Three repetitions per extract were made. The 4-mm diameter mycelial pellets inoculum were collected from 10-days-old *P. infestans* culture using a sterilized cork borer and then placed in the center of each well on the microplate. The plates were incubated at 18 ± 1 °C for three days and the growth of the *P. infestans* strain was measured for each modality (control, DMSO control, extracts, and fractions). The percentage of inhibition was obtained using the following formula: Pi (%) = [(Dm−dm)/Dm] × 100; Dm = average of D1 + D2 + D3 [filament length (mm) of mycelial growth in the DMSO control], and dm = average of d1 + d2 + d3 [filament length (mm) of mycelial growth with extract]. Pi = percent reduction in mycelial growth.

The antifungal activity of the crude extract and fractions against *Z. tritici* was assessed in 12-well microplates containing PDA medium that was either amended or not with compounds at 1000, 500, 250, 125, 62.5, and 31.5 mg·L^−1^, according to the protocol described previously by Platel and coll. (2021) [40]. The fungal growth was recorded eleven days after microplate inoculation with fungal spore suspension by measuring the perpendicular diameter of each fungal colony. The percentage of inhibition was calculated following the same formula used above for *P. infestans*. Dose-response curves for both *P. infestans* and *Z. tritici* were performed on GraphPad Prism software version 9 (GraphPad Software Inc., San Diego, CA, USA). The bioassays for both pathogens were performed twice in two independent experiments with three biological repetitions each.

## 4. Conclusions

Ethnopharmacological and molecular networking approaches have provided good visibility, not only on the chemical composition of the *E. exsul* plant, but also highlighted its importance both in traditional medicine and in the search for bioactive natural substances. In total, 20 compounds were identified through GNPS, and their identity was further confirmed by previous works. The plant extract fractions, in particular F3, were active against the targeted phytopathogens, especially against *P. infestans*. The observed strong antioxidant activity (F8, F4, and F6) reinforces the importance of valorizing this plant in the human health area. Although this study represents the first exploration of chemistry of *E. exsul*, the results are very encouraging and pave the way for further analyses, and a possible isolation to characterize the compounds responsible for the observed biological activities.

## Figures and Tables

**Table 2:** Annotated compounds on the molecular networking of *E. exsul* ethanolic extract.

*m/z* [M+H]^+^	Molecular Formulas [M]	Adducts Types	Cosine Score	Compound
758.570	C_42_H_82_NO_8_P	[M+H]^+^	0.92	1-Palmitoyl-2-linoleoyl-sn-glycero-3-phosphocholine
784.588	C_44_H_80_NO_8_P	[M+H]^+^	0.77	1,2-Dilinoleoyl-sn-glycero-3-phosphocholine
772.582	C_43_H_85_NO_8_P	[M+H]^+^	0.70	1-Heptadecanoyl-2-(9Z-octadecenoyl)-sn-glycero-3-phosphocholine
275.201	C_19_H_30_O_2_	[M+H−H_2_O]^+^	0.73	Androstenediol
309.207	C_19_H_30_O_3_	[M+H]^+^	0.76	Epioxandrolone
321.242	C_21_H_34_O_2_	[M+H]^+^	0.73	Methasteron
454.919	C_24_H_36_O_8_	[M+H]^+^	0.71	[(1*S*,2*R*,4a*R*,8a*R*)-1-Acetyloxy-1,4a-dimethyl-6-oxo-7-propan-2-ylidene-2,3,4,5,8,8a-hexahydronaphthalen-2-yl]-3_acetyloxy-2-hydroxy-2-methylbutanoate
439.840	C_21_H_22_O_9_	[M+Na]^+^	0.78	Isoliquiritin
437.341	C_24_H_32_O_6_	[M+Na]^+^	0.73	[1*S*,3*R*,3a*S*,4*S*,8a*R*)-1-Acetyloxy-3-hydroxy-6,8a-dimethyl-3-propan-2-yl-1,2,3a,4,5,8-hexahydroazulen-4-yl]4-hydroxybenzoate
406.259	C_20_H_30_O_6_	[M+K]^+^	0.81	3-[2-(6,7-Dihydroxy-1,2,4a-trimethylspiro [3,4,6,7,8,8a-hexahydro-2H-naphthalene-5,2′-oxirane]-1-yl)ethyl]-2-hydroxy-2H-furan-5-one
369.263	C_27_H_46_O	[M+H−H_2_O]^+^	0.82	Cholesterol
410.258	C_22_H_32_O_7_	[M+H]^+^	0.74	[6,10a-dihydroxy-4-(hydroxymethyl)-4,7,11b-trimethyl-9-oxo-1,2,3,4a,5,6,6a,7,11,11a-decahydronaphtho[2,1-f][1]benzofuran-5-yl] acetate
507.232	C_32_H_30_N_2_O_4_	[M+H]^+^	0.83	Asperphenamate
269.083	C_16_H_12_O_4_	[M+H]^+^	0.87	Formononetin
413.380	C_27_H_42_O_3_	[M+H]^+^	0.83	3-Oxocholest-4-en-26-oic acid
579.177	C_26_H_28_O_15_	[M+H]^+^	0.76	Rhoifolin
663.463	C_34_H_48_O_13_	[M+H]^+^	0.73	Sarmentoside B

**Table 3:** Antioxidant properties of the crude extract and fractions (F1–F8) of *Erismadelphus exsul*.

Test Sample	FRAP (µM TE/g DW)	DPPH (µM TE/g DW)
EE crude extract	2173.40 ± 13.92	29.04 ± 6.01
EE fraction 1	234.37 ± 12.21	42.47 ± 4.19
EE fraction 2	1959.24 ± 7.33	39.58 ± 1.95
EE fraction 3	1369.14 ± 83.57	145.51 ± 6.69
EE fraction 4	2173.40 ± 7.33	253.67 ± 50.50
EE fraction 5	2246.52 ± 44.42	498.03 ± 23.17
EE fraction 6	2307.54 ± 48.40	464.62 ± 8.57
EE fraction 7	2329.42 ± 47.90	565.51 ± 9.29
EE fraction 8	2187.22 ± 85.49	574.03 ± 7.94
Ascorbic Acid	631.99 ± 39.08	1261.79 ± 150.68

The results are expressed as mean ± standard deviation (S.D.), EE = *Erismadelphus exsul*, DW = dry weight, TE = Trolox-Equivalent.

## Data Availability

Not applicable.

## Supplementary Materials

The following supporting information can be downloaded at: https://www.mdpi.com/article/10.3390/plants11111505/s1, Figure S1: The molecular network of the ethanolic crude extract of Erismadelphus exsul obtained with the GNPS platform and visualized with the Cytoscape 3.9.0 software; Figure S2: Structures and names of compounds identified on the molecular network; Figure S3: Focus on the cluster of antimicrobial ions of interest contained in fractions F1 and F3; Figure S4: Mass spectra of the [M+H]^+^ ions of Mauritine F, Mauritine A, Mauritine A N-oxide, 8-Dihydroantidesmone, 8-Deoxoantidesmone, and Antidesmone obtained by LC–ESI–MS; Table S1: In vitro colony diameters (mm) obtained using two independent experiments for Phytophthora infestans and Zymoseptoria tritici at different concentrations of crude extract and eight fractions from Erismadelphus exsul bark.
