# Supplementary material for: Combined LC-MS/MS and Molecular Networking Approach Reveals Antioxidant and Antimicrobial Compounds from Erismadelphus exsul Bark
Source: Plants (Basel). 2022 Jun 3;11(11):1505. doi: 10.3390/plants11111505 (PMC9182967; doi:10.3390/plants11111505)
Supplement: Supplementary file 1 [file plants-11-01505-s001.zip › plants-1737748-supplementary.pdf]

Supporting materials

This is the supporting information for "Combined LC-MS/MS and molecular networking approach reveals antioxidant and antimicrobial compounds from *Erismadelphus exsul bark*" including global molecular network data, antimicrobial activity and LC-MS spectra.

Contents

Figure S1 : The molecular network of the ethanolic crude extract of *Erismadelphus exsul* obtained with the GNPS platform and visualized with the Cytoscape 3.9.0 software.

Figure S2 : Structures and names of compounds identified on the molecular network

Table S1 : *In vitro* antimicrobial activity : *Phytophthora infestans* and *Zymoseptoria tritici*

Figure S3 : Focus on the cluster of antimicrobial ions of interest contained in fractions F1 and F3.

Figure S4 : Mass spectra of the [M+H]+ ions of Mauritine F, Mauritine A, Mauritine A *N*-oxide, 8-Dihydroantidesmone, 8-Deoxoantidesmone, and Antidesmone obtained by LC–ESI–MS.

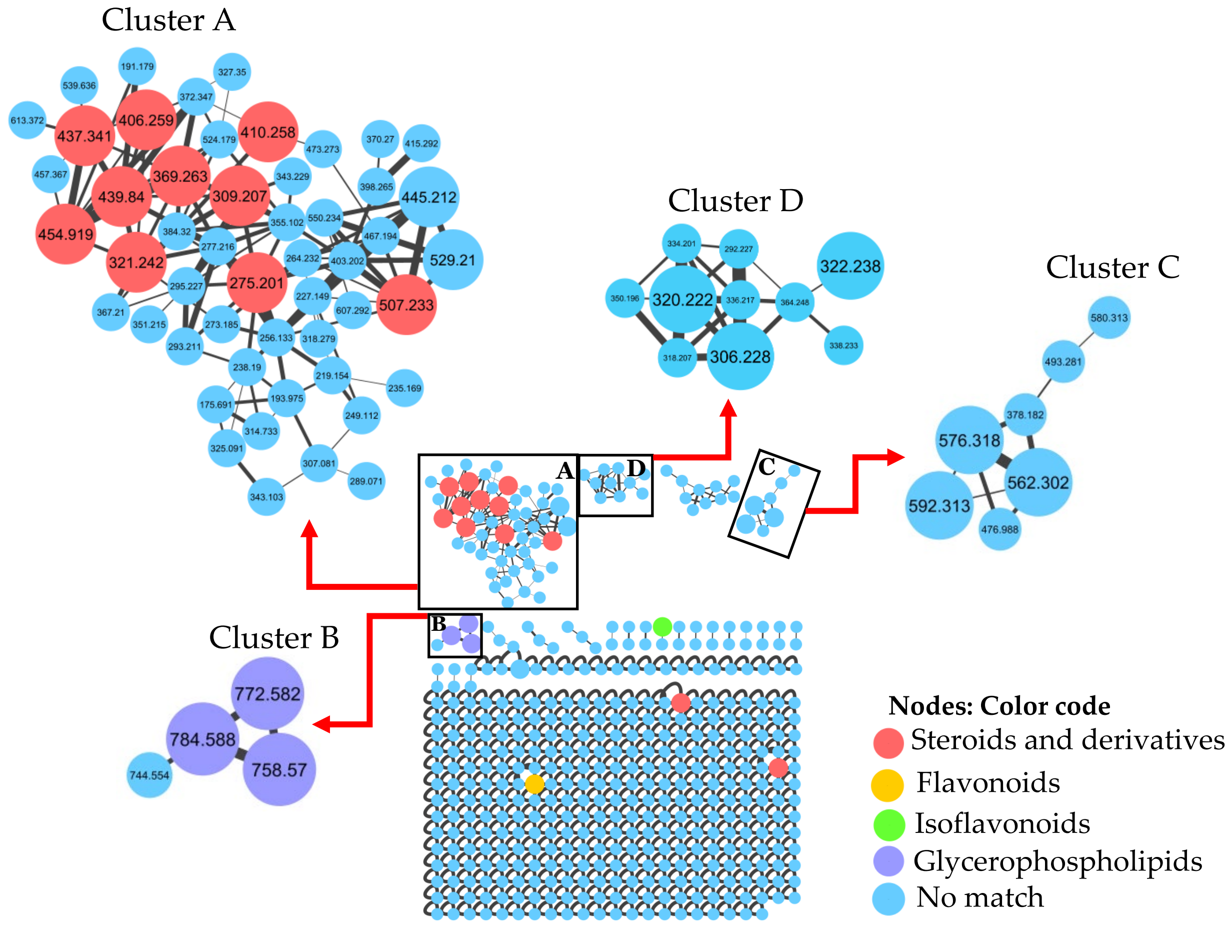

Figure S1. The molecular network of the ethanolic crude extract of *Erismadelphus exsul* obtained with the GNPS platform and visualized with the Cytoscape 3.9.0 software. The resulting analysis and parameters for the network can be accessed via this link :
http://gnps.ucsd.edu/ProteoSAFe/status.jsp?task=f4b18f2a813c440bab3cf78abe5aa648.

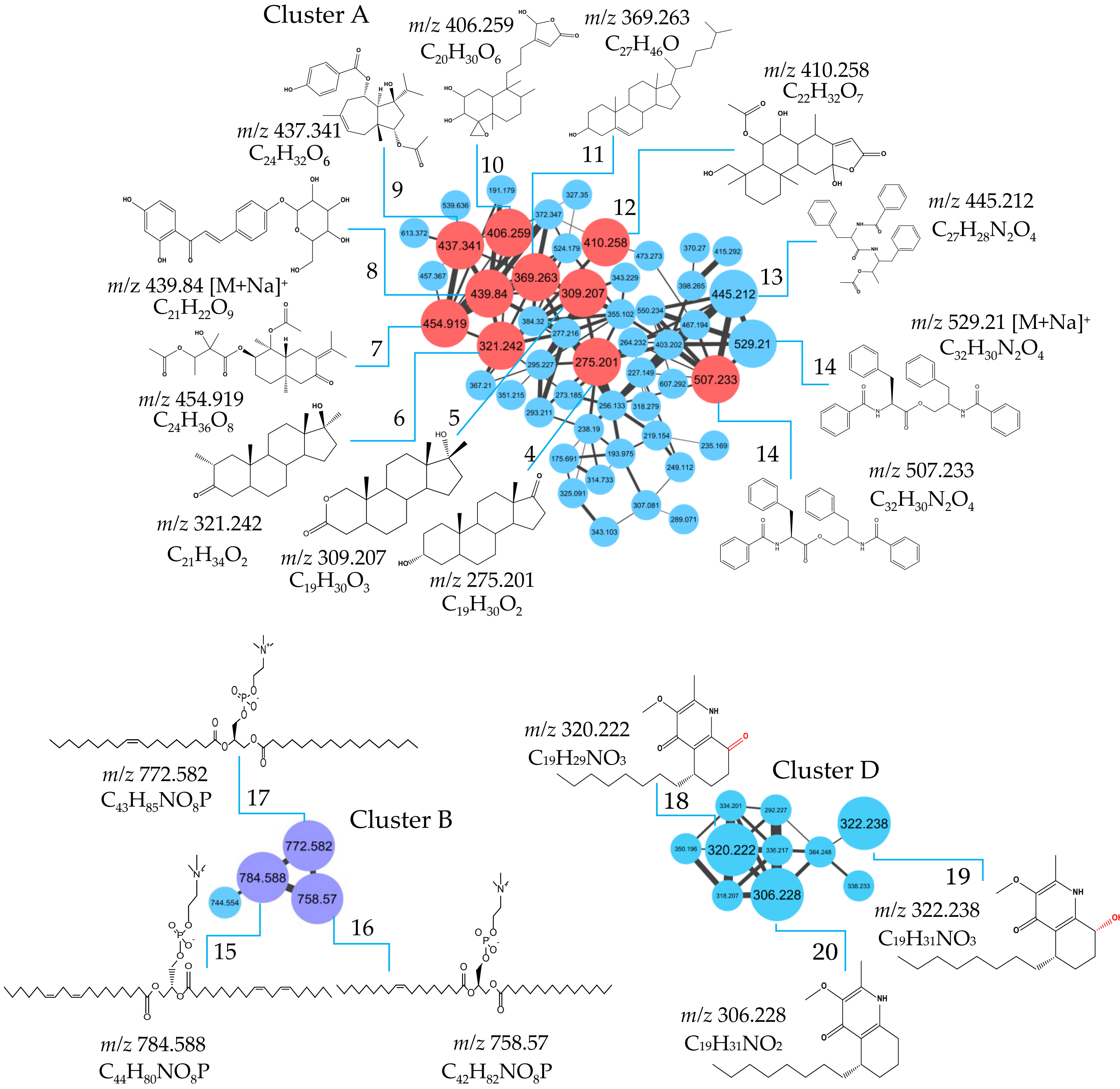

Figure S2. Shows the structures of the compounds identified on the molecular network **(4)** h_127_1_Androstenediol, **(5)** h_61_17_Epioxandrolone, **(6)** Methasteron, **(7)** [(1S,2R,4aR,8aR)-1-Acetyloxy-1,4a-dimethyl-6-oxo-7-propan-2-ylidene-2,3,4,5,8,8a-hexahydronaphthalen-2-yl]-3_acetyloxy-2-hydroxy-2-methylbutanoate, **(8)** Isoliquiritin, **(9)** [1S,3R,3aS,4S,8aR)-1-Acetyloxy-3-hydroxy-6,8a-dimethyl-3-propan-2-yl-1,2,3a,4,5,8-hexahydroazulen-4-yl]4-hydroxybenzoate, **(10)** 3-[2-(6,7-Dihydroxy-1,2,4a-trimethylspiro[3,4,6,7,8,8a-hexahydro-2H-naphthalene-5,2′-oxirane]-1-yl)ethyl]-2-hydroxy-2H-furan-5-one, **(11)** Cholesterol, **(12)** [6,10a-dihydroxy-4-(hydroxymethyl)-4,7,11b-

trimethyl-9-oxo-1,2,3,4a,5,6,6a,7,11,11a-decahydronaphtho[2,1-f][1]benzofuran-5-yl] acetate, **(13)** Asperglaucide, **(14)** Asperphenamate, **(15)** 1,2-Dilinoleoyl-sn-glycero-3-phosphofcholine, **(16)** 1-Palmitoyl-2-linoleoyl-sn-glycero-3-phosphocholine, **(17)** 1-Heptadecanoyl-2-(9Z-octadecenoyl)-sn-glycero-3-phosphocholine, Antidesmone (**18**), 8-Dihydroantidesmone (**19**) and 8-Deoxoantidesmone (**20**).

Table S1. *In vitro* colony diameters (mm) obtained using two independent experiments for *Phytophthora infestans* and *Zymoseptoria tritici* at different concentrations of crude extract and eight fractions from *Erismadelphus exsul* bark.

	**Concentration (mg.L^{-1})**													
	**0**		**31.5**		**62.5**		**125**		**250**		**500**		**1000**	
	**Rep1**	**Rep2**	**Rep1**	**Rep2**	**Rep1**	**Rep2**	**Rep1**	**Rep2**	**Rep1**	**Rep2**	**Rep1**	**Rep2**	**Rep1**	**Rep2**
Phytophthora infestans														
**DMSO**	**13.3** (0.2)	**13.3** (0.3)	**13.5** (1.3)	**13.5** (0.5)	**13.2** (0.3)	**13.2** (1.3)	**12.7** (1.0)	**12.8** (1.2)	**11.7** (2.4)	**12.3** (1.5)	**10.3** (3.0)	**10.2** (2.0)	**10.8** (2.1)	**10.0** (1.8)
**Crude extract**	**13.3** (0.2)	**13.3** (0.3)	**9.7** (2.8)	**9.5** (2.2)	**7.0** (3.0)	**7.2** (1.3)	**6.8** (2.3)	**7.5** (1.5)	**6.0** (2.5)	**4.8** (1.2)	**3.3** (1.9)	**2.2** (3.8)	**2.3** (4.0)	**2.3** (4.0)
**F1**	**13.3** (0.2)	**13.3** (0.3)	**12.2** (1.0)	**12.0** (1.0)	**9.5** (1.8)	**10.7** (2.3)	**8.0** (1.8)	**8.0** (3.6)	**3.5** (2.8)	**6.7** (2.4)	**1.0** (1.7)	**1.0** (1.7)	**0.7** (1.2)	**0.7** (1.2)
**F2**	**13.3** (0.2)	**13.3** (0.3)	**10.8** (2.8)	**11.7** (1.8)	**9.7** (2.8)	**9.0** (3.1)	**7.3** (2.5)	**7.8** (2.8)	**6.2** (2.8)	**7.5** (2.6)	**3.0** (3.6)	**2.0** (3.5)	**0.7** (1.2)	**1.3** (1.5)
**F3**	**13.3** (0.2)	**13.3** (0.3)	**9.7** (2.9)	**10.0** (1.7)	**9.3** (1.8)	**7.8** (1.5)	**8.7** (2.4)	**7.0** (0.5)	**4.5** (1.5)	**4.2** (4.0)	**1.0** (1.7)	**1.8** (3.2)	**0.0** (0.0)	**0.0** (0.0)
**F4**	**13.5** (1.1)	**14.3** (0.4)	**11.5** (3.9)	**12.0** (1.0)	**10.5** (3.0)	**10.5** (1.3)	**9.8** (2.9)	**8.7** (0.6)	7.5 (2.5)	**8.3** (1.6)	**6.2** (1.0)	**4.5** (1.5)	**3.7** (3.3)	**1.0** (0.0)
**F5**	**13.5** (1.1)	**14.3** (0.4)	**12.2** (1.9)	**11.8** (1.0)	**10.0** (2.6)	**10.8** (0.6)	**9.7** (0.23)	**9.8** (2.0)	**8.2** (1.4)	**9.3** (2.4)	**0.67** (1.2)	**7.7** (1.6)	**3.7** (3.5)	**2.3** (2.3)
**F6**	**13.5** (1.1)	**14.3** (0.4)	**11.7** (3.6)	**13.2** (3.5)	**9.8** (2.1)	**12.3** (3.8)	**10.2** (2.3)	**11.8** (1.0)	**8.2** (3.3)	**10.5** (1.3)	**4.8** (3.5)	**9.2** (0.8)	**6.0** (3.8)	**4.5** (1.3)
**F7**	**13.5** (1.1)	**14.3** (0.4)	**11.3** (2.9)	**13.8** (0.8)	**9.5** (4.0)	**12.0** (1.8)	**10.2** (3.3)	**12.2** (1.0)	**8.7** (3.3)	**10.0** (1.7)	**7.0** (5.3)	**7.2** (2.0)	**4.7** (4.1)	**3.8** (3.5)
**F8**	**13.5** (1.1)	**14.3** (0.4)	**11.8** (2.9)	**12.7** (1.9)	**10.8** (4.2)	**11.8** (1.0)	**9.5** (3.3)	**11.2** (1.4)	**7.5** (3.9)	**9.5** (1.8)	**7.3** (2.0)	**7.8** (2.5)	**4.5** (4.0)	**6.7** (1.5)
Zymoseptoria tritici														
**DMSO**	**9.8** (0.5)	**9.9** (0.2)	**9.0** (0.0)	**9.5** (0.7)	**9.3** (0.6)	**9.2** (0.3)	**9.8** (0.3)	**9.2** (0.3)	**9.3** (0.3)	**9.3** (0.3)	**9.5** (0.5)	**8.8** (0.3)	**8.8** (0.6)	**9.5** (0.7)
**Crude extract**	**9.8** (0.5)	**9.9** (0.2)	**7.8** (0.3)	**7.7** (0.3)	**7.0** (0.0)	**7.5** (0.0)	**6.8** (0.3)	**6.8** (0.3)	**6.2** (0.3)	**6.2** (0.3)	**5.8** (0.4)	**5.7** (0.3)	**5.0** (0.0)	**5.0** (0.0)
**F1**	**9.8** (0.5)	**9.9** (0.2)	**8.8** (0.3)	**10.0** (0.0)	**9.5** (0.5)	**9.0** (0.0)	**9.2** (0.3)	**9.5** (0.5)	**9.3** (0.3)	**9.2** (0.3)	**9.0** (0.0)	**8.8** (0.3)	**8.7** (0.3)	**8.7** (0.6)
**F2**	**9.8** (0.5)	**9.9** (0.2)	**8.0** (0.0)	**8.2** (0.3)	**7.0** (0.0)	**6.7** (0.6)	**6.2** (0.3)	**6.8** (0.3)	**6.0** (0.0)	**6.0** (0.0)	**5.0** (0.0)	**5.0** (0.0)	**3.8** (0.3)	**5.0** (0.0)
**F3**	**9.8** (0.5)	**9.9** (0.2)	**6.7** (0.3)	**7.0** (0.0)	**6.0** (0.0)	**6.0** (0.0)	**5.0** (0.0)	**5.3** (0.6)	**5.0** (0.0)	**5.0** (0.0)	**4.0** (0.0)	**4.0** (0.0)	**3.0** (0.0)	**3.5** (0.0)
**F4**	**9.8** (0.5)	**9.9** (0.2)	**8.3** (0.3)	**7.0** (0.0)	**7.0** (0.0)	**6.0** (0.0)	**6.3** (0.3)	**5.7** (0.6)	**6.0** (0.0)	**5.0** (0.0)	**5.0** (0.0)	**4.0** (0.0)	**4.5** (0.5)	**3.3** (0.3)
**F5**	**9.8** (0.5)	**9.9** (0.2)	**8.0** (0.0)	**7.3** (0.6)	**7.0** (0.0)	**6.8** (0.3)	**6.0** (0.0)	**6.0** (0.0)	**5.3** (0.6)	**5.7** (0.6)	**5.3** (0.3)	**5.0** (0.0)	**5.0** (0.0)	**4.7** (0.3)

**F6**	**9.8** (0.5)	**9.9** (0.2)	**8.3** (0.6)	**8.0** (0.0)	**7.0** (0.0)	**6.8** (0.3)	**6.3** (0.3)	**6.0** (0.0)	**6.0** (0.0)	**6.0** (0.0)	**5.3** (0.6)	**5.3** (0.6)	**5.0** (0.0)	**4.5** (0.5)
**F7**	**9.8** (0.5)	**9.9** (0.2)	**7.8** (0.3)	**6.2** (0.8)	**6.8** (0.3)	**7.2** (0.3)	**6.0** (0.0)	**6.0** (0.0)	**5.7** (0.3)	**5.3** (0.6)	**5.0** (0.0)	**4.8** (0.3)	**5.0** (0.0)	**5.0** (0.0)
**F8**	**9.8** (0.5)	**9.9** (0.2)	**7.8** (0.3)	**7.5** (0.5)	**6.8** (0.3)	**7.0** (0.0)	**6.3** (0.3)	**6.0** (0.0)	**6.0** (0.0)	**6.0** (0.0)	**5.8** (0.3)	**5.7** (0.3)	**5.2** (0.3)	**5.8** (0.3)

Rep 1: Repetition 1 ; Rep2: Repetition 2

Values in bold indicate means of *in vitro* colony diameters measured at three and eleven days after microplate inoculation with *P. infestans* and *Z. tritici*, respectively, while the values in brackets indicate the corresponding standard deviations obtained with three biological replicates for each tested concentration.

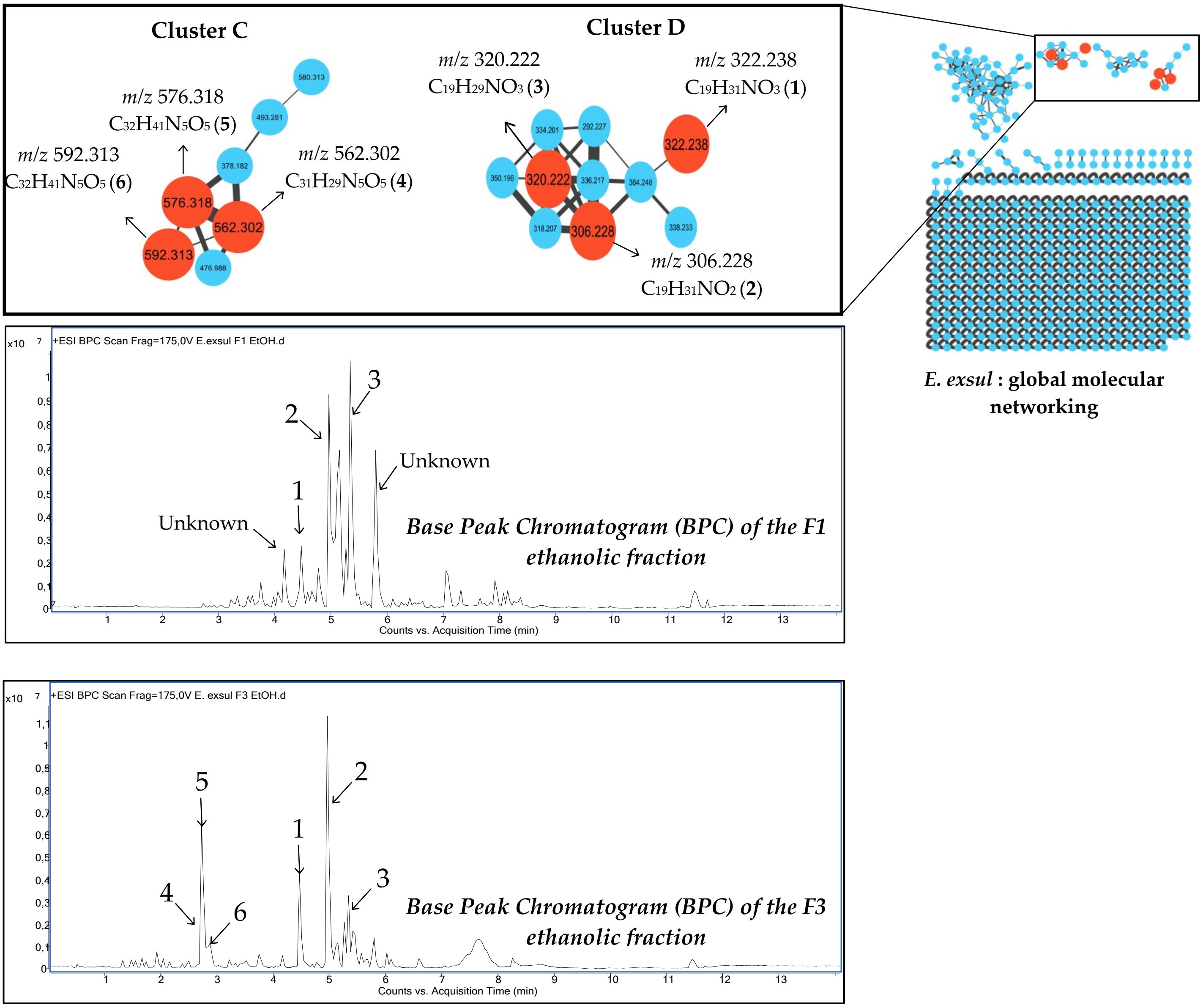

Figure S3. Focus on the cluster of antimicrobial ions of interest contained in fractions F1and F3.

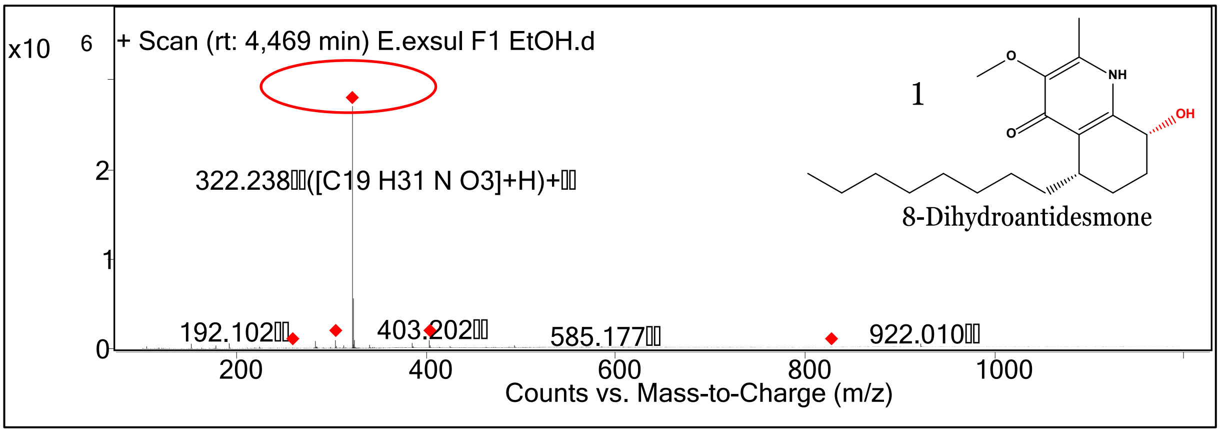

x10 6
+ Scan (rt: 4,469 min) E.exsul F1 EtOH.d
322.238 ([C19 H31 N O3]+H)+
192.102
403.202
585.177
922.010
0
1
2
200
400
600
800
1000
Counts vs. Mass-to-Charge (m/z)
1
O
NH
OH
8-Dihydroantidesmone

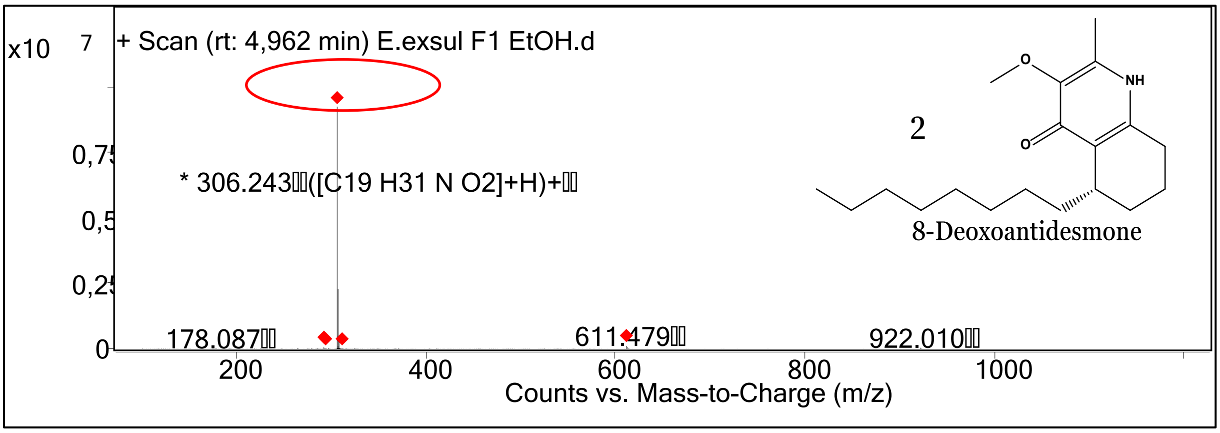

x10 7
+ Scan (rt: 4,962 min) E.exsul F1 EtOH.d
* 306.243 ([C19 H31 N O2]+H)+
178.087
611.479
922.010
0
0,25
0,5
0,75
200
400
600
800
1000
Counts vs. Mass-to-Charge (m/z)
2
O
NH
8-Deoxoantidesmone

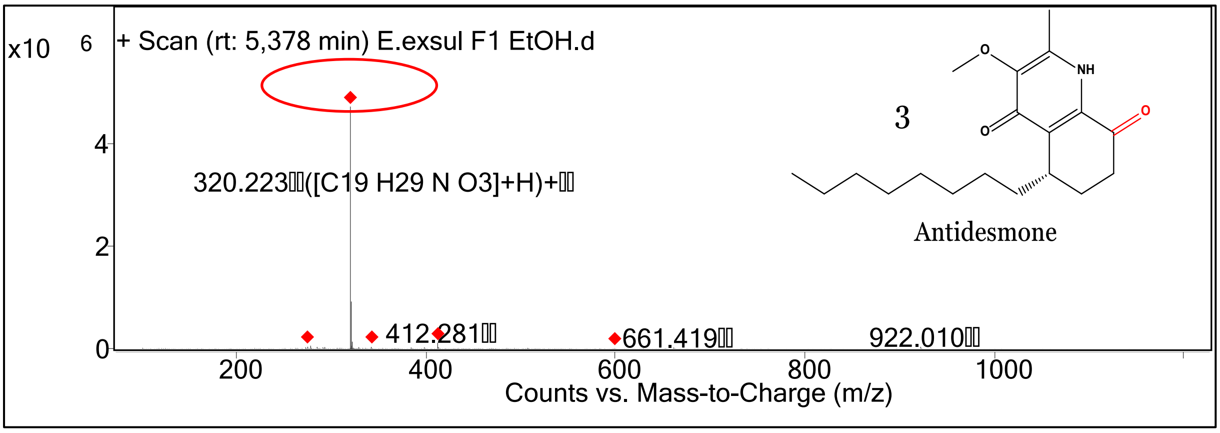

x10 6
+ Scan (rt: 5,378 min) E.exsul F1 EtOH.d
320.223 ([C19 H29 N O3]+H)+
412.281
661.419
922.010
0
2
4
200
400
600
800
1000
Counts vs. Mass-to-Charge (m/z)
3
O
NH
Antidesmone

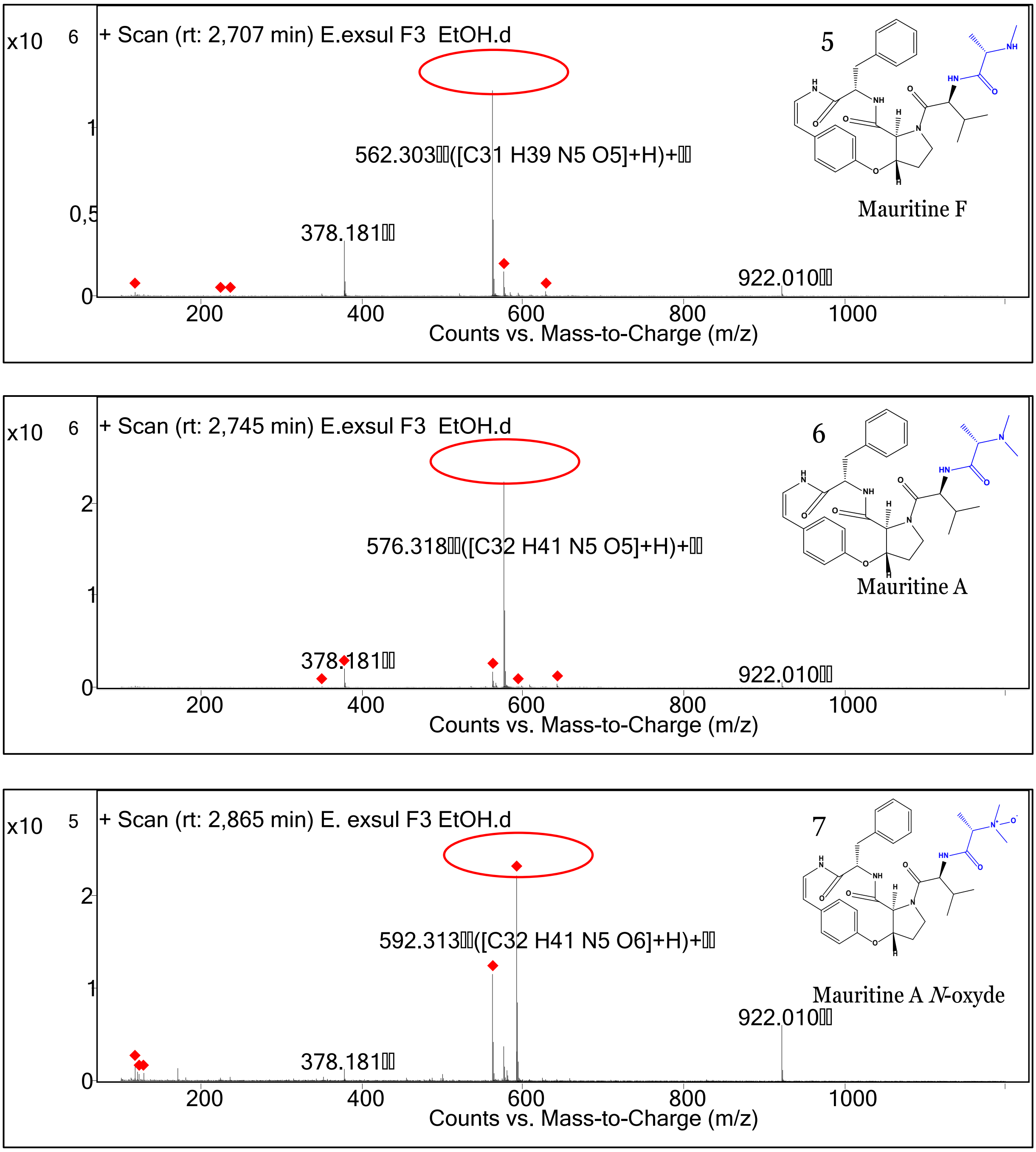

Figure S4. Mass spectra of the $[M+H]^+$ ions of Mauritine A , Mauritine F , Mauritine A N-oxide, 8-Dihydroantidesmone (2), 8-Deoxoantidesmone (3), and Antidesmone (4) obtained by LC–ESI–MS.
